# 32.1 MECHANISMS OF ABNORMAL POSTTRANSLATIONAL PROTEIN PROCESSING IN SCHIZOPHRENIA BRAIN

**DOI:** 10.1093/schbul/sby014.132

**Published:** 2018-04-01

**Authors:** James Meador-Woodruff

**Affiliations:** University of Alabama at Birmingham

## Abstract

**Background:**

Molecular disturbances of neurotransmitter systems have long been held to be a core feature of the pathophysiology of schizophrenia. Despite years of study of neurotransmitter associated protein expression at multiple levels of gene expression, reports of abnormal neurotransmitter receptor transcript, protein, and signaling complex expression in schizophrenia brain have often been conflicting. These inconsistencies led us to reconsider neurotransmitter-based hypotheses of schizophrenia not as a problem of receptor number, or as a defect of neurotransmitter systems, but rather as a dysregulation of central cellular processes regulating the intracellular distribution of signaling proteins. Our working hypothesis is that a fundamental dysregulation of intracellular processes exists in schizophrenia, resulting in abnormal assembly, trafficking, and intracellular targeting of many key proteins involved in neurotransmission and other critical cellular functions. Previous studies have shown the important roles posttranslational lipid and carbohydrate modifications play in targeting receptors, transporters, and other proteins between intracellular compartments and the synapse, and in the lateral translocation of such molecules between lipid microdomains at the distal end of forward trafficking pathways. Accordingly, we have predicted that abnormal posttranslational lipid and/or sugar modification of proteins by occurs in schizophrenia. We have previously reported changes in extent of N-linked glycosylation as well as of the lipid modifications palmitoylation and N-myristoylation on target proteins in schizophrenia brain. In an ongoing project to elucidate mechanisms of these changes, we have studied expression patterns of key enzymes associated with these posttranslational modifications.

**Methods:**

Using well characterized samples of postmortem brain from schizophrenia and matched comparison subjects, we assayed transcript expression of enzymes associated with posttranslational protein modifications by lipids and carbohydrates using microarrays and qPCR. Next, we assayed protein expression of a subset of enzymes using western blot analyses. To determine the brain cell specificity of protein changes, we used laser capture microdissection (LCM) of neuronal and glial cells to harvest specific cell populations from postmortem brains, and developed and validated a capillary electrophoresis system for ultra-low quantity protein concentration (the ProteinSimple WES system) to measure protein expression within LCM harvested cells.

**Results:**

Using microarray and qPCR, multiple transcript changes were found in schizophrenia cortex. The most substantial number of altered transcripts were found for those encoding enzymes associated with multiple aspects of posttranslational carbohydrate modifications of proteins, including N-acetylglucosaminyltransferases (GlcNAcTs), glucosyltransferases, glucosidases, N-acetylgalactosaminyltransferases (GalNAcTs), galactosyltransferases, mannosidases, fucosyltransferases, fucosidases, sialyltransferases, and sialidases. Fewer changes were found for lipid modification enzymes.

Subsequently, protein expression of candidate proteins associated with these posttranslational modifications were determined by western blot analyses. Changes in protein expression of mutlple enzymes associated with glycan modification of proteins were found in schizophrenia. These include significant decreases in expression of the N-acetylglucosaminyltransferases B3GNT8 and MGAT4A, and the fucosyltransferase FUT8. Increased protein expression was found for the fucosyltransferase POFUT2, the sialyltransferase ST8SIA2, the glucosyltransferase UGGT2, and the mannosidase EDEM2. Numerous protein changes were also found in enzymes associated with lipid and glycolipid modifications. These include decreased expression of the prenylation associate denzyme subunits farnesyl-protein transferase α-subunit (FNTA), geranylgeranyltransferase type I β-subunit (PGGT1B), and rab geranylgeranyltransferase β-subunit (RABGGTB). Glycophosphatidylinositol (GPI)-anchor attachment 1 protein (GPAA1) is increased in these subjects.

To determine the cell-specific pattern of these protein changes, we have developed an LCM-capillary electrophoresis assay to isolate protein from LCM harvested cells to allow multiplex protein quantification in 500 ng of protein obtained from these cells. We have validated that we can reliably harvest cortical neuronal subtypes and astroglia, are able to measure 4 proteins simultaneously in samples from these cells lines, and are currently collecting cells to extend these findings into cell-specific studies to determine if the changes we have found in posttranslational modification proteins are widely specific or specific to given subpopulations of brain cells.

**Discussion:**

These data support our earlier findings of altered patterns of the posttranslational modifications of both glycosylation and lipid modification of proteins in the cortex of schizophrenia. By identifying changes in both mRNA and protein expression of key enzymes associated with these posttranslational modifications, we have begun to elucidate potential mechanisms of these earlier observations.

One of the challenges that has plagued schizophrenia research for decades is that many different neurotransmitter and neurochemical systems have been implicated and studied in this illness, and reconciling this large literature is challenging. These many changes in numerous different systems suggest, however, that rather than schizophrenia being a disorder of a given neurotransmitter system, it is rather a disturbance of core intracellular processes that underlie regulation of multiple neurochemical systems. The machinery associated with posttranslational modifications of proteins is a possible substrate that could reconcile prior abnormalities identified in myriad systems. We have proposed that a fundamental defect in the brains of those affected with this illness is abnormal assembly, trafficking and receptor dynamics of many different proteins in schizophrenia that is due mechanistically to abnormal posttranslational modifications that influence intracellular targeting and trafficking of proteins between subcellular compartments. Dysregulation of lipid and glycan modification of proteins are likely candidates for such a process, and these present data begin to elucidate the mechanisms from which these abnormalities occur.

